# EntrezAJAX: direct web browser access to the Entrez Programming Utilities

**DOI:** 10.1186/1751-0473-5-6

**Published:** 2010-06-21

**Authors:** Nicholas J Loman, Mark J Pallen

**Affiliations:** 1 Centre for Systems Biology, University of Birmingham, Edgbaston, Birmingham, B15 2TT, UK

## Abstract

Web applications for biology and medicine often need to integrate data from Entrez services provided by the National Center for Biotechnology Information. However, direct access to Entrez from a web browser is not possible due to 'same-origin' security restrictions. The use of "Asynchronous JavaScript and XML" (AJAX) to create rich, interactive web applications is now commonplace. The ability to access Entrez via AJAX would be advantageous in the creation of integrated biomedical web resources. We describe EntrezAJAX, which provides access to Entrez eUtils and is able to circumvent same-origin browser restrictions. EntrezAJAX is easily implemented by JavaScript developers and provides identical functionality as Entrez eUtils as well as enhanced functionality to ease development. We provide easy-to-understand developer examples written in JavaScript to illustrate potential uses of this service. For the purposes of speed, reliability and scalability, EntrezAJAX has been deployed on Google App Engine, a freely available cloud service. The EntrezAJAX webpage is located at http://entrezajax.appspot.com/

## Background

**Figure 1:**
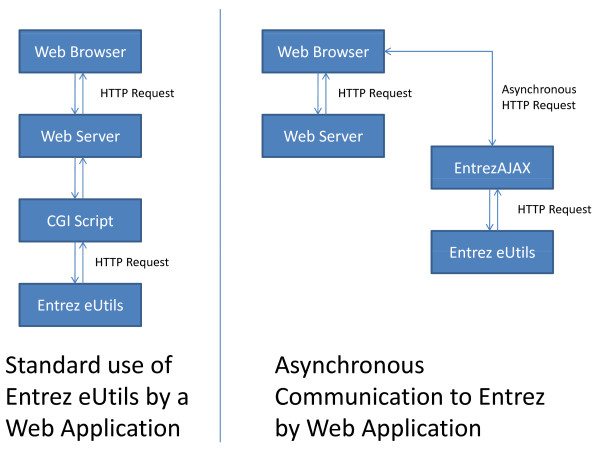
**Anatomy of an AJAX Request**. A typical web application which requires access to Entrez eUtils uses a synchronous pattern as shown on the left. EntrezAJAX permits an asynchronous pattern which does not couple the request to Entrez eUtils to the web server serving the original request.

Web applications for biology and medicine often need to integrate data from external data sources. Entrez, provided by the National Center for Biotechnology Information (NCBI) provides a searchable interface to important biological databases including PubMed, GenBank and GenPept [1]. Application developers may perform searches of Entrez directly by accessing the publically available Entrez Programming Utilities (Entrez eUtils) interface [2]. However, until now there has been no API available which allows web application ('webapp') developers to access Entrez eUtils directly from the browser. Developers are limited to accessing Entrez eUtils through software code running on the web "backend". This approach is less than ideal as it requires the call to Entrez eUtils to have been completed before each web page can be rendered. This means that that pages load slowly and may become blocked if the Entrez eUtils interface is unavailable for some reason (e.g. downtime, network congestion). It also means bandwidth required to provide the service may be increased due to the overhead of fetching the page from the backend and returning it to the user. This synchronous approach may also result in larger pages being generated, which also slow down page loading (Figure 1).

The use of "Asynchronous JavaScript and XML" (AJAX) has transformed the usability and power of webapps over the past 5 years. These technologies have been instrumental in the development of rich web applications. Many popular web services, including Google, Twitter and Flickr permit direct programmatic access to their databases and services by offering AJAX APIs [2-4]. The availability of such APIs has given the ability to integrate multiple data sources within a web application without the need for backend server code. The ease-of-use of such approaches has driven the adoption of 'mash-up' applications which make it simple to integrate datasets from a diversity of sources. A typical example is the ability to link location data from status messages in a Twitter feed with a map display from Google to give a geographical display of the dataset. Although biomedical resources have slowly begun to embrace the use of AJAX to improve the user's experience [5-7], they rarely offer AJAX APIs for direct browser access to databases.

We describe EntrezAJAX , an AJAX service which provides fast, convenient and reliable access to Entrez eUtils from any web browser. By circumventing the web browser security restrictions this API can be used by any developer wishing to incorporate Entrez results into their webapp.

## Implementation

The essence of the EntrezAJAX implementation is a web proxy-like service backend, which is able to convert results from Entrez eUtils to JavaScript Object Notation (JSON).

On grounds of security, web browsers are configured so that they do not allow a site to connect to a second, unrelated site. This is known as the 'same-origin' policy. Typically, if data providers wish to allow third-party websites access to their data through the browser they provide specific JavaScript APIs served from their web domain. These APIs give the browser implicit permission to request further data from that domain. In order to circumvent the same-origin policy requirement we support JSON with padding (JSONP) which takes advantage of the web browser's ability to load JavaScript source from arbitrary remote locations [8].

The backend was built using the model-view-controller (MVC) web framework Django [9]. MVC approaches to web development help enforce good development practice by separating data (model) from presentation (view) and business logic (controller) in code. We used the Entrez module of Biopython [10] to access Entrez eUtils.

The backend software was deployed on the web using Google App Engine (GAE) [11], a freely-available cloud computing service. The Datastore and Memcache components of GAE were used to store the registry of developer API keys and cache search results respectively.

JavaScript developer examples were written to demonstrate common uses for the service. These example codes utilise the jQuery JavaScript library [12] to make AJAX calls.

The results of web requests are stored in a temporary memory cache for 24 hours. This value is configurable on a per-application basis. Each request is given a key, which comprises the method name and the alphabetically sorted parameter list (excluding the developer API key). Each request's key is checked against the cache first before contacting Entrez eUtils. If the key is present in the cache, the result is returned directly from the cache. This acts to reduce the time taken to serve requests and to reduce the number of calls made to Entrez eUtils to save bandwidth.

## Results

Developers wishing to use EntrezAJAX must first register their website on the project homepage to receive a developer API key. Web developers make requests to EntrezAJAX by constructing a URL consisting of three components; the endpoint, the method name and a dictionary of parameters (a hash of key/value pairs). The parameter dictionary must include the developer API key, which identifies the originator of the request. Other parameters are method-dependent. Each of the Entrez eUtils applications (EInfo, ESearch etc.) are exposed as a separate method name.

Developers wishing to access the Entrez eUtils 'Esearch' application should construct a URL in the following format, substituting <APIKEY>:

http://entrezajax.appspot.com/esearch?apikey=<APIKEY>&db=pubmed&term=Crick+AND+Watson

In order to accommodate common patterns of usage, combined methods permit two calls to Entrez eUtils to be chained together. In these cases, the first of these methods return a list of GI numbers, which are not returned to the user but are instead passed to the second method via the id parameter. Table 1 lists the available methods.

**Table 1:** List of EntrezAJAX Method Calls

eUtils Passthrough Calls	Combined calls
efetch	esearch+esummary
einfo	esearch+efetch
elink	esearch+elink
espell	elink+esummary
esummary	elink+efetch

List of the available method calls from EntrezAJAX

Results are always returned as JSON-format strings. Such strings can be natively deserialized into JavaScript objects. This process is fast and avoids the need for any specific parsing code (e.g. XML parsing) to be implemented in JavaScript. JSON is widely supported by other languages either natively or through third-party libraries. Languages with JSON support available include Python, Java and Perl.

To support JSONP and to bypass the same site policy, all method calls support an optional parameter callback. The value of this parameter is used to specify the name of the JavaScript function, which will be called with the JSON string as its first argument.

We anticipate developers will use a JavaScript helper library such as jQuery, Prototype [13] or YUI [14] to perform AJAX tasks. We developed our example code using the jQuery scripting language as this provides native support for JSONP callbacks and is simple and easy to read.

NCBI specify strict limitations on the use of the Entrez Programming Utilities service on a per-developer basis. Where practical, we have enforced these limitations in code. The service will not permit more than three requests to be passed through to Entrez within one second. Additionally, the tool and email parameters are automatically filled-in using the information supplied when registering for a developer API key. We urge users of this service to familiarise themselves with the NCBI limitations and ensure their application meets them.

The EntrezAJAX project website has example code for using EntrezAJAX. These include the retrieval of results from PubMed and GenBank, retrieval of journal articles related to a nucleotide sequence and the ability to automatically correct users' spelling. Additionally, EntrezAJAX is heavily used on the authors' own xBASE resource for comparative bacterial genomics [15].

## Discussion

### Google App Engine Platform

The use of GAE has significant advantages for implementation of services such as EntrezAJAX. These include the availability of a large in-memory cache, persistent data storage, a distributed network infrastructure and automatic failover mechanisms. During the development of EntrezAJAX we did not experience any occasion when the service was not available. However, the application sometimes took several seconds to respond, probably because a new GAE process was started up.

Currently any user may deploy an application on GAE for free. However, the application must stay within certain limits otherwise the application may be prevented from serving further requests until the quota period has elapsed. Quotas are subject to change, but important limits to consider when implementing this service include the incoming HTTP request limit, the UrlFetch limit and the Memcache API limit. The limitations imposed by the free tariff we believe are sufficient to cater for likely demand for the service in the near future. However we plan to monitor the service usage in case limits are reached. In that case, heavy users of the service will be contacted and we may suggest that they deploy the EntrezAJAX application from their own Google App Engine account and update their endpoint details accordingly.

### Dependency on Entrez eUtils

The EntrezAJAX service is dependent on the availability of NCBI eUtils to work correctly. If NCBI eUtils is unavailable, requests will not be fulfilled, unless the request is already stored in the cache.

### Access to Other Services

EntrezAJAX provides a working implementation for providing direct web browser access to biomedical resources accessible via the web. Therefore, we encourage users wishing to access other resources via AJAX to contribute code accordingly. However we believe this intelligent proxy approach represents a stepping-stone along the path to more integrated biomedical resources on the web. We are actively looking for other bioinformatics web resources that would benefit from a similar interface to EntrezAJAX. We also hope this project will inspire developers to invest the time and energy in producing AJAX-compatible endpoints for their databases.

## Availability and Requirements

Project name: EntrexAJAX

Project home page: http://entrezajax.appspot.com/

Source code home page: http://github.com/nickloman/entrezajax

Operating system(s): Platform-independent

Programming language: Python 2.5 +

Other requirements: Django 1.1 +, BioPython 1.53 +, Google App Engine

License: Apache License, Version 2.0

Any restrictions to use by non-academics: None

## List of Abbreviations

API: Application Programming Interface; HTTP: HyperText Transfer Protocol

## Competing interests

The authors declare that they have no competing interests.

## Authors' contributions

NJL conceived and implemented the software. NJL and MJP jointly drafted the manuscript. Both authors have read and approved the final manuscript.
